# Diagnostic Accuracy of USG and MRI for the Detection of Rotator Cuff Injury

**DOI:** 10.7759/cureus.68199

**Published:** 2024-08-30

**Authors:** P Madhavi, Prakash Patil

**Affiliations:** 1 Department of Radiodiagnosis, Krishna Vishwa Vidyapeeth (Deemed to be University), Karad, IND

**Keywords:** rotator cuff tear, systematic review, diagnostic accuracy, magnetic resonance imaging, ultrasound

## Abstract

Introduction: Rotator cuff disease frequently causes shoulder pain and is diagnosed using various radiological methods alongside history and physical examination. Arthrography has traditionally been employed for this purpose, but newer non-invasive techniques such as ultrasonography (USG) and magnetic resonance imaging (MRI) are increasingly used. However, no single method is universally agreed upon as the best diagnostic tool, each having its own limitations.

Objectives: To evaluate how effectively ultrasound and MRI can diagnose rotator cuff tears.

Materials and methods: Seventy patients suspected of having a rotator cuff tear underwent investigations at the Radiology Department of Krishna Vishwa Vidyapeeth (Deemed to be University), Karad. USG and MRI examinations were done on the same day, along with a detailed history. USG was conducted using a GE LOGIQ P9 machine with a high-frequency 3-12 MHz transducer. MRI was conducted using a 1.5T Siemens Magnetom Avanto scanner.

Results: Pain and stiffness are the most common complaints in rotator cuff tears. The predisposing factors include male predominance, increasing age, dominant hand use, and trauma history. The supraspinatus tendon is the most frequently injured, with partial tears, especially articular surface tears, being more common than full-thickness tears. Clinical examinations, USG, and MRI are valuable in diagnosing rotator cuff tears.

Conclusion: Our findings indicate that USG may not be as reliable in detecting rotator cuff tears as once believed. A positive ultrasound result is more trustworthy than a negative one. In contrast, MRI demonstrates greater sensitivity and overall diagnostic accuracy compared to both ultrasonography and clinical assessment for detecting rotator cuff tears.

## Introduction

Shoulder pain is a frequent complaint with various potential causes, including rotator cuff tears, adhesive capsulitis, bursitis, arthritis, tendinitis, and fractures. Symptoms can develop gradually or suddenly after activities like heavy lifting or trauma. Physical examination and plain radiographs can be used to check for bone and soft tissue abnormalities, but they do not provide evidence of acute rotator cuff tears.

Conventional invasive arthrography is increasingly being replaced by non-invasive MRI and ultrasound. Arthrography is used for patients who are contraindicated to MRI or when ultrasound results are unclear [1]. Ultrasound imaging of the shoulder is quick, cost-effective, and allows for real-time evaluation of dynamic movements, making it a practical initial choice for assessing tendons and soft tissues. However, it has limitations, including operator dependence, an inability to identify bone pathologies, and the necessity of radiological anatomy and awareness of scan artifacts.

MRI provides detailed joint information essential for the diagnosis and treatment decisions. During a shoulder MRI, the patient lies with the arm in lateral rotation, and a surface coil is used for signal detection. T2-weighted sequences with fat suppression, proton density fat-saturated (PDFS) or short tau inversion recovery (STIR) sequences, are valuable for diagnosing rotator cuff pathologies [2]. The examination typically includes axial, coronal, and sagittal planes. MRI can reveal the dimensions, depth, thickness, and shape of rotator cuff tears, involvement of adjacent structures, muscle atrophy [3], and details about the coracoacromial arch and impingement, directly implying treatment and prognosis.

Although MRI is more expensive and time-consuming than ultrasound, it offers high-quality images of shoulder muscles and ligaments, making it useful in case of inconclusive ultrasound or when trained personnel are not available [4].

## Materials and methods

An observational study was meticulously designed for comparing ultrasound to MRI to evaluate their effectiveness in diagnosing rotator cuff injuries. The study spanned an 18-month period from July 2022 to December 2023, taking place at the Department of Radiodiagnosis, Krishna Vishwa Vidyapeeth, Karad. Prior to its commencement, the study received ethical clearance to ensure that it complied with all ethical guidelines, prioritizing patient safety and research integrity.

The inclusion criterion was patients aged 20-80 years who were suspected of having rotator cuff injuries and exclusion criteria were those with claustrophobia, metallic implants, cardiac pacemakers, or any cochlear implants due to potential interference with imaging or patient safety concerns.

For imaging, the study utilized two primary modalities: ultrasound and MRI. The ultrasound examinations were performed by the same operator using a GE LOGIQ P9 machine (GE Healthcare, Chicago, IL) equipped with a 3-12 MHz transducer. This high-frequency transducer allowed for detailed imaging of the shoulder's soft tissues. The ultrasound protocol included a comprehensive evaluation of the rotator cuff tendons, the acromioclavicular joint (ACJ), and the joint cavity. Dynamic shoulder examinations were conducted to assess for impingement issues, where the shoulder was moved through various positions to reveal potential abnormalities that might not be visible in static imaging. The protocol accepted was first positioning the patient either sitting or lying down with the shoulder exposed. For assessing the biceps tendon, the arm is in a neutral position, the elbow is flexed and the forearm supinated, the subscapularis arm is externally rotated, the supraspinatus arm is internally rotated like picking the wallet from back pocket, and the infraspinatus arm is extended to the opposite shoulder. Longitudinal and transverse views were obtained.

**Figure 1:**
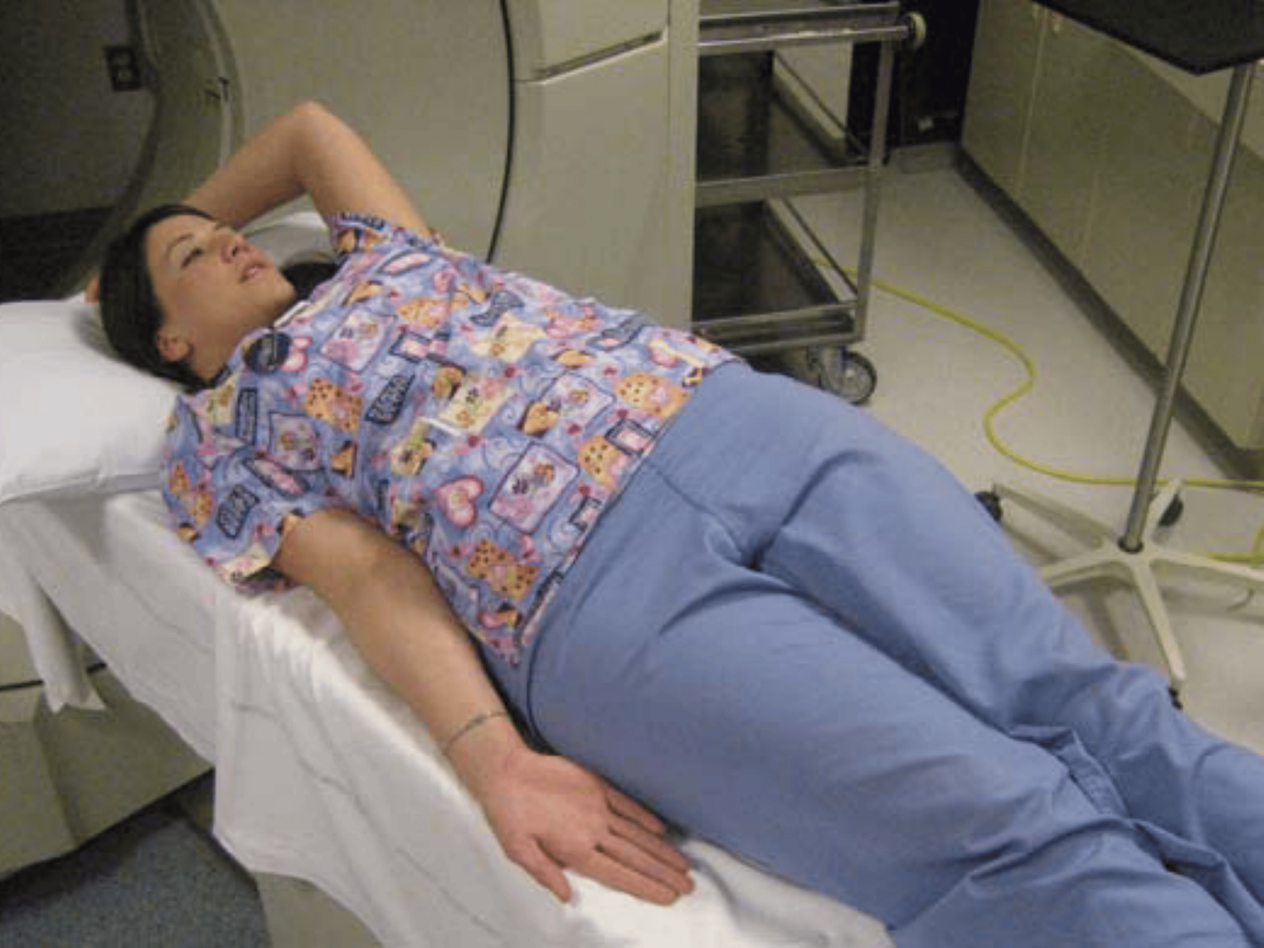
Patient position for MRI shoulder Adapted from AHI MSK CT Protocols (https://xanatomy.net/CTPImages/Shoulder-position.png)

The patient position for recording MRI shoulder is shown in Figure 1. 

MRI was performed using a 1.5T Siemens Magnetom Avanto scanner (Siemens Healthineers, Erlangen, Germany), known for its high-resolution imaging capabilities. A dedicated surface coil was employed to enhance image quality specific to the shoulder. The imaging protocol involved positioning the patient supine with the affected shoulder externally rotated to optimize the visualization of the rotator cuff structures. The MRI scans were taken with a slice thickness of 4 mm, which strikes a balance between detail and coverage, and field of view settings were 150 mm for sagittal and axial images and 180 mm for coronal images. This setup ensured a detailed and comprehensive imaging of the shoulder.

Data collected from these imaging modalities were meticulously entered into Microsoft Excel 2007 (Microsoft, Redmond, WA) and analyzed using IBM SPSS Statistics, version 28 (IBM SPSS, Armonk, NY). The results were charted in tables. The statistical method used was the chi-square test for examining the relationship between categorical variables, such as the presence or absence of rotator cuff injuries as detected by each imaging modality. Diagnostic test validity was assessed through several key metrics: sensitivity (the ability to correctly identify those with the injury), specificity (the ability to correctly identify those without the injury), positive predictive value (the likelihood that a positive test result accurately indicates an injury), negative predictive value (the likelihood that a negative result accurately indicates no injury), and overall diagnostic accuracy (the proportion of true results, both positive and negative, among all test results).

To assess the agreement between the two diagnostic tests (ultrasound and MRI), Kappa statistics were applied. This measure helps determine how well the results from one test correlate with those from the other, beyond what would be expected by chance alone. The study's findings were visually represented using tables, which facilitated a clear and comprehensive understanding of the results. These visual aids were crucial for summarizing data, illustrating trends, and presenting the diagnostic performance of the imaging modalities in a straightforward manner.

## Results

The age of the patients ranged from 20 to 80 years, with a mean of 47.5 years. The patients involved in the study were divided into three age groups: <40 years, 41-50 years, and >50 years. The majority of rotator cuff injures were observed after 50 years of age in 43% of the subjects, 30% at 41 to 50 years, and 26% at <40 years as described in Table 1.

**Table 1:** Distribution of cases according to age N: Number of cases; %: Percentage; yrs: Years

AGE	N	%
<40 yrs	19	26.7
41 to 50 yrs	21	30.0
>50 yrs	30	43.3
Total	70	100.0

In patients aged older than 50 years, 37.1% show tears as compared to those younger than 50 years, where 30% of the patients show tears in the rotator cuff tendons. In patients older than 50 years of age, five showed tendinosis and in those younger than 50 years, seven showed tendinosis as described in Table 2.

**Table 2:** Tendon abnormalities according to age Yrs: Years

	Age			Total
	<40 yrs	41 to 50 yrs	>50 yrs	
Normal	9	2	0	11
Tendinosis	5	2	5	12
Partial tear	2	12	14	28
Full thickness	2	5	12	19
Total	18	21	31	70

Most of the patients were right-handed (87.1%) and all left-handed patients had rotator cuff injuries. Among the right-handed patients, 80.3% had injuries on their right side, as shown in Table 3.

**Table 3:** Distribution of the dominant hand according to the affected side

Dominant hand	Affected side		Total
	Left	Right	
Left	9	0	9
Right	12	49	61
Total	21	49	70

The most common presentation is pain (47.1%). The second most common presentation is the inability to do overhead abduction (24.2%) as described in Table 4.

**Table 4:** Distribution of cases according to clinical data N: Number of cases; %: Percentage

Clinical data	N	%
Pain	33	47.1
Stiffness	7	10.0
Inability to do overhead abduction	17	24.2
Pain and stiffness	9	13.3
Weakness	2	3.3
Pain and weakness	2	3.3
Total	70	100.0

The number of patients with a history of trauma (28.5%) is listed in Table 5. 

**Table 5:** Distribution of cases according to a history of trauma N: Number of cases; %: Percentage

Trauma	N	%
Absent	50	71.4
Present	20	28.5
Total	70	100.0

In USG, the most commonly injured tendon is supraspinatus muscle followed by subscapularis. Tendinosis is most commonly seen in supraspinatus muscle as described in Table 6.

**Table 6:** Tendon injuries according to USG findings SS: Supraspinatus; IP: Infraspinatus; SUB: Subscapularis; TM: Teres Minor; BT: Biceps Tendon

Tendons	SS	IP	SUB	TM	BT
No tear	39	68	61	70	70
Articular surface partial tear	9	1	5	0	0
Bursal surface partial tear	5	0	4	0	0
Full thickness tear	7	1	0	0	0
Intrasubstance tear	5	0	0	0	0
Tendinosis	5	0	0	0	0
Total	70	70	70	70	70

In MRI, the most commonly injured tendon is the supraspinatus muscle followed by subscapularis. Tendinosis is most commonly seen in supraspinatus muscle as described in Table 7.

**Table 7:** Tendon injuries according to MRI findings SS: Supraspinatus; IP: Infraspinatus; SUB: Subscapularis; TM: Teres Minor; BT: Biceps Tendon

Tendons	SS	IP	SUB	TM	BT
No tear	19	68	56	70	70
Articular surface partial tear	23	1	9	0	0
Bursal surface partial tear	5	0	0	0	0
Full thickness tear	7	1	5	0	0
Intrasubstance tear	2	0	0	0	0
Tendinosis	14	0	0	0	0
Total	70	70	70	70	70

USG findings demonstrated lower sensitivity in detecting tendon injuries across all sites, with the highest sensitivity observed in detecting injuries to the subscapular tendon. The agreement between USG and MRI findings, assessed by Kappa, was strongest for subscapular tears as described in Table 8. 

**Table 8:** Validity of USG findings with MRI findings in tendon injuries SS: Supraspinatus; IP: Infraspinatus; SUB: Subscapularis; TM: Teres Minor; BT: Biceps Tendon

	Sensitivity	Specificity	Positive Predictive Value	Negative Predictive Value	Diagnostic Accuracy	Kappa Degree of Agreement
SS	59%	100%	100%	47%	70%	0.33
IP	50%	100%	100%	97%	97%	0.46
SUB	67%	100%	100%	92%	93%	0.74
TM	-	100%	-	100%	100%	-
BT	-	100%	-	100%	100%	-

In USG, two cases were detected to have calcification in supraspinatus tendon as shown in Table 9.

**Table 9:** Calcification diagnosed by USG N: Number of cases; %: Percentage

	N	%
Supraspinatus	2	2.8
Infraspinatus	0	0.0
Subscapularis	0	0.0
Teres minor	0	0.0
Biceps tendon	0	0.0

In MRI, two cases were detected to have calcification in supraspinatus tendon as shown in Table 10.

**Table 10:** Calcification diagnosed by MRI N: Number of cases; %: Percentage

	N	%
Supraspinatus	2	2.8
Infraspinatus	0	0.0
Subscapularis	0	0.0
Teres minor	0	0.0
Biceps tendon	0	0.0

A 100% accuracy was noted for identifying calcifications in USG and MRI in rotator cuff injuries as shown in Table 11. 

**Table 11:** Validity of USG findings with MRI findings in detecting calcification SS: Supraspinatus; IP: Infraspinatus; SUB: Subscapularis; TM: Teres Minor; BT: Biceps Tendon

	Sensitivity	Specificity	Positive Predictive Value	Negative Predictive Value	Diagnostic Accuracy
SS	100%	100%	100%	100%	100%
IP	-	100%	-	100%	100%
SUB	-	100%	-	100%	100%
TM	-	100%	-	100%	100%
BT	-	100%	-	100%	100%

MRI detected 37 patients positive for peribicipital tendon fluid (PTF) whereas USG detected 35 cases out of 70, but missed two cases. The p-values for both tests are approximately <0.001 as described in Table 12. There was a notable correlation between the USG and MRI results.

**Table 12:** Association between USG findings and MRI findings in detecting peribicipital tendon fluid PTF: Peribicipital Tendon Fluid; USG: Ultrasound; MRI: Magnetic Resonance Imaging

PTF in USG	PTF in MRI		Total	p value
	Absent	Present		
Absent	33	2	35	<0.001*
Present	0	35	35	
Total	33	37	70	

MRI shows SA-SD bursal fluid in 35 cases, while USG detected 33 cases and missed two. MRI is superior in detecting SA-SD bursal fluid. MRI detected SC bursal fluid in 24 out of 70 cases, while USG identified 20 cases and missed four. The p-values for both tests are approximately <0.001 as shown in Table 13. This indicates a statistically significant relationship between both types of bursal fluid in USG and MRI findings.

**Table 13:** Association between USG findings and MRI findings in detecting bursal fluid SA-SD: Subacromial-subdeltoid; SC: Subcoracoid; USG: Ultrasound; MRI: Magnetic Resonance Imaging

		MRI Findings		Total	p value
		Absent	Present		
Subacromial-subdeltoid bursal fluid (SA-SD) in USG	Absent	35	2	37	<0.001*
	Present	0	33	33	
	Total	35	35	70	
Subcoracoid bursal fluid (SC) in USG	Absent	46	4	50	<0.001*
	Present	0	20	20	
	Total	46	24	70	

The p-value for the test of independence between joint effusion in USG and joint effusion in MRI is approximately 0.000021 as described in Table 14. This indicates a strong evidence against the null hypothesis of independence, suggesting a statistically significant relationship between these two variables. This indicates that MRI outperforms USG in detecting joint effusion.

**Table 14:** Association between USG findings and MRI findings in detecting joint effusion USG: Ultrasound; MRI: Magnetic Resonance Imaging

Joint Effusion in USG	Joint Effusion in MRI		Total	p value
	Absent	Present		
Absent	33	7	40	0.000021
Present	0	30	30	
Total	33	37	70	

MRI showed 21 positive cases of acromio-clavicular joint hypertrophy (ACJH) and USG detected 21 cases of ACJH out of 70, signifying that USG equals MRI in detecting ACJH as demonstrated in Table 15.

**Table 15:** Association between USG findings and MRI findings in detecting acromio-clavicular joint hypertrophy ACJH: Acromio-clavicular joint hypertrophy; USG: Ultrasound; MRI: Magnetic resonance imaging

ACJH in USG	ACJH in MRI		Total	p value
	Absent	Present		
Absent	49	0	49	0.000021
Present	0	21	21	
Total	49	21	70	

MRI identified 12 cases of subacromial (SA) impingement out of 70, while USG detected seven cases and missed five. This significant association indicates that MRI is more effective than USG in detecting SA impingement. Similarly, MRI detected two cases of subcoracoid (SC) impingement out of 70, and USG also detected two cases as shown in Table 16. The significant association here shows that USG is equivalent to MRI in detecting SC impingement.

**Table 16:** Association between USG and MRI in detecting impingement lesions SA: Subacromial; SC: Subcoracoid; USG: Ultrasound; MRI: Magnetic resonance imaging

		MRI Findings		Total
		Absent	Present	
Subacromial impingement (SA) in USG	Absent	58	5	63
	Present	0	7	7
	Total	58	12	70
Subcoracoid impingement (SC) in USG	Absent	68	0	68
	Present	0	2	2
	Total	68	2	70

In our study, USG demonstrated 100% specificity for all except SA impingement. USG showed the highest sensitivity for diagnosing ACJH and SA-SC impingement, with the lowest sensitivity for detecting joint fluid. Diagnostic accuracy was 100% for ACJH and SC impingement. Kappa statistics: PTF, bursal SA-SD: 0.50 (moderate agreement), bursal SC: 0.42 (moderate agreement), bursal joint effusion: 0.36 (fair agreement), bursal ACJH: 1.00 (perfect agreement), impingement SA: 0.38 (fair agreement), impingement SC: 1.00 (perfect agreement) as described in Table 17.

**Table 17:** Accuracy of USG with MRI in detecting fluid, ACJH, and impingement PTF: Peribicipital tendon fluid; SA-SD: Subacromial-subdeltoid; SC: Subcoracoid; ACJH: Acromio-clavicular joint hypertrophy; SA: Subacromial; PPV: Positive predictive value; NPV: Negative predictive value

		Sensitivity	Specificity	PPV	NPV	Diagnostic Accuracy	Kappa Degree of Agreement
	PTF	94%	100%	100%	94%	97%	0.5
Bursal	SA-SD	94%	100%	100%	94%	97%	0.5
	SC	83%	100%	100%	92%	94%	0.42
	Joint Effusion	81%	100 %	100%	82%	90%	0.36
	ACJH	100%	100%	100%	100%	100%	1.00
Impingement	SA	100%	92 %	100%	92%	93%	0.38
	SC	100%	100%	100%	100%	100%	1.00

The rotator cuff injury was detected in 20% of the patients with labral tear as described in Table 18.

**Table 18:** Distribution of labral tear N: Number of cases; %: Percentage

Labral Tear	N	%
Absent	56	80
Present	14	20
Total	70	100

There is a fair agreement between USG and MRI, as shown in Table 19. 

**Table 19:** Comparison of MRI diagnosis with USG diagnosis USG: Ultrasound; MRI: Magnetic resonance imaging

	Sensitivity	Specificity	Positive Predictive Value	Negative Predictive Value	Diagnostic Accuracy	Kappa Degree of Agreement
USG vs MRI	67%	100%	100%	43%	73%	0.44

When the final diagnosis is compared with surgery,MRI demon strated the highest diagnostic accuracy as shown in Table 20. 

**Table 20:** Comparison of final diagnosis between MRI and USG USG: Ultrasound; MRI: Magnetic Resonance Imaging

	Sensitivity	Specificity	Positive Predictive Value	Negative Predictive Value	Diagnostic Accuracy	Kappa Degree of Agreement
MRI versus Final diagnosis	92%	80%	96%	67%	90%	0.66
USG versus Final diagnosis	64%	100%	100%	36%	70%	0.37

## Discussion

In a prospective study at Krishna Vishwa Vidyapeeth, Karad, 70 patients with suspected rotator cuff injuries were evaluated with both ultrasound (USG) and MRI following comprehensive clinical assessments. While MRI offers superior multiplanar imaging capabilities compared to CT, providing detailed visualization and precise localization of rotator cuff abnormalities, it is costly and less suitable as an initial diagnostic tool. In contrast, USG, being non-invasive and more affordable, serves well as a primary investigation method.

According to age

In our study, patients aged 20 to 80 years (mean age 47.5 years) showed a higher incidence of injuries among those older than 50 years (43.3%). Needell et al. [5] found higher tendinosis rates in younger individuals and more tears in older age groups, consistent with our observation of tears being more prevalent in those older than 50 years (37.1%) compared to those younger than 50 (30%), and tendinosis being more common in the younger group.

Association between the dominant and affected side

Urwin et al. [6] predicted a higher prevalence of shoulder disorders, noting that rotator cuff tears are more common in the dominant arm. In our study, 87.1% of the patients were right-handed and 12.8 % were left-handed. Left-handers (100%) had rotator cuff injuries on the left side and 80.3% of the right-handers had injuries on the right side. This indicates that there is a significant association between dominant hand use and the affected side.

Clinical presentation

Pain is the most common symptom associated with rotator cuff injury, commonly felt in the anterior, superior, and lateral areas of the shoulder. It worsens during overhead movements or abduction activities such as combing hair or lifting against resistance.

Weakness may result from pain inhibition or muscular fatigue, with significant weakness often hindering arm elevation above shoulder level. Stiffness can arise from pain or rotator cuff weakness. In our study, pain was the most frequent complaint (47.1%) among patients with rotator cuff disorders, consistent with established literature [7] highlighting rotator cuff issues as a primary cause of shoulder pain.

Association with trauma

In our study, 20 (28.5%) patients had a history of trauma. Among these, 16 patients had rotator cuff tears, indicating that trauma is a predisposing factor for rotator cuff tears, consistent with the existing literature [8,9].

Comparison of USG and MRI findings in tendon Injuries

Supraspinatus tendon is most commonly affected, observed in 26 patients, followed by the subscapularis in nine patients and the infraspinatus in two patients. Conversely, no cases of teres minor or biceps tendon involvement were noted. These findings align with Jerosch et al.'s research [10], which analyzed shoulder specimens from 122 patients and reported that 78% of cases had isolated supraspinatus involvement, with no tears occurring without supraspinatus tendon involvement. Similarly, De Palma [11], in their study of 96 cadaver shoulders, observed that the supraspinatus tendon was most frequently affected, with tear incidence and severity increasing with age. In our study, the most commonly involved tendon was the supraspinatus, affecting 37 patients, followed by the subscapularis in 14 patients, and the infraspinatus in two patients. No cases of teres minor or biceps tendon involvement were observed. USG had missed 11 cases of supraspinatus and five cases of subscapularis tears.

Comparison of USG findings with MRI results in tendon injuries

USG showed varying sensitivity in detecting tendon injuries: 59% for supraspinatus, 50%for infraspinatus, and 67% for subscapularis. But its specificity was uniformly high at 100% across all sites. Subscapularis tears had the highest agreement (Kappa = 0.76). These findings are consistent with Martín-Hervás et al.'s [12] study, highlighting USG's lower sensitivity compared to MRI in diagnosing rotator cuff tears despite high specificity.

Comparison of USG with MRI for the detection of calcification

In our study, calcification of the supraspinatus tendon was detected in two out of 70 patients (2.8%) by both USG and MRI, indicating that both imaging modalities are equally effective for identifying tendon calcification.

Comparison of USG with MRI in the detection of tendon fluid

In our study, MRI detected peribicipital tendon fluid (PTF) in 52.8% of the patients (37 out of 70), whereas USG identified it in 50% (35 out of 70) of the patients. MRI demonstrated superior sensitivity in detecting PTF compared to USG. Additionally, PTF was observed in 37 patients (53.33%) in our study. Among these, 28 patients (75.6 %) had tears, five (12.5%) had tendinosis, and four (10.8%) had a normal tendon. These results align with Beall et al.'s [13] findings, indicating a significant association of tendon fluid and rotator cuff tears in the supraspinatus and subscapularis components.

Comparison of USG with MRI in detecting bursal fluid and joint effusion

MRI identified subacromial-subdeltoid (SA-SD) bursal fluid in 35 patients (50%), whereas USG detected it in 33 cases (47.1%) but missed two. For subcoracoid (SC) bursal fluid, MRI detected it in 24 patients (34.2%), whereas USG found it in 20 cases (28.5%) and missed four. In terms of joint effusion, MRI detected it in 37 patients (52.8%), while USG identified it in 30 cases (42.8%) and missed seven. Overall, MRI demonstrated higher effectiveness in detecting bursal fluid and joint effusion compared to USG, as indicated by significant associations (p < 0.0001) between their findings. In our study, joint effusion was present in 37 patients (52.8 %). Among those with joint effusion, 30 (81.08%) had cuff tears, two (5.40%) had tendinosis, and five (13.5%) had a normal tendon. Hollister et al. [14] found that 52% of the patients with surgically confirmed rotator cuff tears had fluid in the joint, bursa, or both, highlighting a strong correlation between such fluid presence and cuff tears. 

Comparison of USG with MRI in the detection of acromio-clavicular joint hypertrophy

MRI detected acromioclavicular joint hypertrophy (ACJH) in 21 patients (30%) and USG confirmed its presence all the 21 cases. Among the 21 patients with ACJH, 16 (76.1%) had a tear, two (9.52 %) had tendinosis, and three (14.2%) had a normal tendon. These findings are consistent with Needel et al.'s study [5], suggesting that ACJH increases with age and is commonly associated with tears.

Comparison of USG with MRI in the detection of impingement

MRI identified 12 cases of subacromial (SA) impingement out of 70, while USG detected seven cases and missed five. This significant association indicates that MRI is more effective than USG in detecting SA impingement. Similarly, MRI detected two cases of subcoracoid (SC) impingement out of 70, and USG also detected two cases. The significant association here shows that USG is equivalent to MRI in detecting SC impingement. This was consistent with Read et al. [15] evaluated ultrasound for preoperative impingement syndrome diagnosis, finding a sensitivity of 67.56%.

Labrum and rotator cuff tears

Tung et al. [16] found that 68% of patients with arthroscopically confirmed labral tears also had rotator cuff tears. In our study of 70 patients, 14 (20%) were found to have labrum tears. 

## Conclusions

In our study, rotator cuff tears were most frequently observed in patients older than 50 years, with a higher incidence in males and a preference for the dominant arm. Trauma was a significant factor, affecting 80% of the cases. Pain was the predominant symptom, reported by 47.1% of the patients, followed by difficulty with overhead abduction. The supraspinatus tendon was most commonly affected, while partial tears were more prevalent than full thickness tears. Among partial tears, articular surface tears were the most common.

Imaging findings showed a significant correlation between peribicipital tendon fluid and rotator cuff tears, with 75% of the patients with this fluid also having rotator cuff injuries. Acromio-clavicular joint hypertrophy was also strongly associated with rotator cuff tears. MRI proved to be more sensitive and accurate than ultrasound for detecting peribicipital tendon fluid, joint effusion, and various bursal fluids. Although both imaging methods were similarly effective in detecting calcifications and joint hypertrophy, MRI alone identified labral tears. Ultrasound had high specificity but lower sensitivity, making it less reliable than MRI, which had superior diagnostic accuracy and sensitivity for detecting rotator cuff tears.
